# Developing an Early Warning System for Financial Networks: An Explainable Machine Learning Approach

**DOI:** 10.3390/e26090796

**Published:** 2024-09-17

**Authors:** Daren Purnell, Amir Etemadi, John Kamp

**Affiliations:** School of Engineering and Applied Science, George Washington University, Washington, DC 20052, USA; etemadi@gwu.edu (A.E.); jckamp2018@email.gwu.edu (J.K.)

**Keywords:** complex systems, macroprudential economics, machine learning, systemic risk

## Abstract

Identifying the influential variables that provide early warning of financial network instability is challenging, in part due to the complexity of the system, uncertainty of a failure, and nonlinear, time-varying relationships between network participants. In this study, we introduce a novel methodology to select variables that, from a data-driven and statistical modeling perspective, represent these relationships and may indicate that the financial network is trending toward instability. We introduce a novel variable selection methodology that leverages Shapley values and modified Borda counts, in combination with statistical and machine learning methods, to create an explainable linear model to predict relationship value weights between network participants. We validate this new approach with data collected from the March 2023 Silicon Valley Bank Failure. The models produced using this novel method successfully identified the instability trend using only 14 input variables out of a possible 3160. The use of parsimonious linear models developed by this method has the potential to identify key financial stability indicators while also increasing the transparency of this complex system.

## 1. Introduction

Identifying evolving threats to financial networks is challenging, necessitating a careful balance between competing considerations. On the one hand, we must acknowledge that the next financial crisis may not occur via the same channels as previous threats to financial stability. Conversely, it would be “irresponsible to ignore what we have learned, should the same thing ever happen again” [1]. Current early warning systems for financial instability fail to adequately capture nonlinear, time-varying relationships between network participants, leading to gaps in predictive accuracy and regulatory utility [2,3,4]. This study aims to address these gaps by developing an explainable machine learning model that incorporates these complex relationships. Utilizing a collection, or ensemble, of machine learning models with other statistical methods enables the identification of variables that may indicate an evolving threat while incorporating information from past financial crises. Balancing the identification of novel vulnerabilities with historical insights is crucial for developing early warning systems that are robust across different financial crisis scenarios. This balance ensures that systems are not overly reliant on past patterns, which may not recur, while still leveraging valuable lessons from historical data to inform future risk assessments.

This challenge is associated with entropy and information theory, focusing on the uncertainty associated with identifying low-probability but highly impactful/high-information events. Financial crisis mitigation is challenging because of the complexity of the system, uncertainty of a failure, and nonlinear, time-varying relationships between network participants. According to Shannon [5], entropy measures the uncertainty in a system, or the information content associated with the probability distribution of the outcomes. Entropy and information theory offer a framework for understanding and quantifying uncertainty and risk in financial systems, which are central to the concepts of financial stability and economics [6,7]. In this study, we implement a novel methodology to select the most influential subset of variables that are capable of efficiently representing financial crises. By introducing a machine learning methodology to capture the risk inherent in this complex system, one can assess the level of unpredictability and potential for disorder within financial markets, thereby gaining insights into the resilience and stability of economic systems.

Financial network models have been used to understand markets and the systemic threats to financial stability arising from the default of an individual or financial institution, rendering it unable to fulfill its financial obligations [8,9,10]. At times, the effects of a default can spread from one network participant to another, resulting in financial contagion. Financial contagion, or the cascading effect whereby an economic shock in one sector of the market propagates to other sectors, can result in the systemic failure of the financial network, which can affect global economies [11,12,13]. The concept of financial contagion is derived from epidemiology and represents the notion that economic shocks can be disseminated among financial institutions, markets, and countries through various channels. This phenomenon lends itself to representation through network models that depict interbank risks as a system of nodes representing financial entities and the relationships or edges that connect them. Debt claims, known as the network channel, interconnect banks with edge values representing the proportion of debt owed between the two parties. The market, known as the liquidity channel, restricts banks when they sell assets to raise cash during periods of financial stress [13]. The inter-network relationships between financial systems can be challenging to identify and interpret, specifically when nonlinear relationships are involved and may not hold over time [3].

This study proposes a method to estimate threats to financial stability that balances the desire to identify novel vulnerabilities while incorporating historical information from past financial crises. We utilize a machine learning (ML) ensemble, SHapley Additive exPlanations (SHAPs) [14], and modified Borda count for variable selection [15,16], together with financial network models to represent the propagation of systemic cascading failures between banks. Our ML ensemble consisted of a sequential neural network (SNN), support vector regression (SVR), extreme gradient-boosted tree, and random forest (RF) models. ML-based methods utilizing SNN [17], SVR [4], and decision tree-based methods [18], such as extreme gradient-boosted trees and random forests (RFs), can capture and represent these complex relationships. Once the most influential variables have been identified, we train an explainable linear model to represent the edge values in the network. The extra step of training a linear model is taken because economics and regulatory policy require explainable and easy-to-use models. ML models have generally been ineffective in terms of these two requirements because of their “black-box” nature [2]. The resulting linear regression model is then integrated into a network representation of the financial system to inform our understanding of the propagation of financial contagion that could result in systemic failure. We validate the proposed methodology by applying it to the March 2023 failure of Silicon Valley Bank through its bank holding company, Silicon Valley Financial.

The contributions of this study are as follows:

- An innovative method to identify novel threats to financial stability that incorporates historical information from past financial crises and captures the complex, uncertain, nonlinear, and time-varying relationships between financial institutions;
- A novel variable selection method based on an ensemble of ML models, SHAP, and a modified Borda count method to train a more parsimonious and explainable linear model that predicts relationship value weights between network participants;
- A case study application validating the utility of the proposed method using data from the March 2023 Silicon Valley Bank Crisis.

## 2. Related Work and Background

Previous studies on financial early warning systems [EWSs] have focused on identifying generalized, statistical, or threshold-based factors that predict financial crises to forewarn decision-makers [19]. This research has traditionally consisted of multivariate regression models in which a relationship is statistically defined between the predicted crisis and a set of explanatory variables. However, other studies have focused on financial signal analysis, where financial statements or other supervisory data are used to identify institutional outliers whose performance exceeds an optimized threshold [20]. Similarly, network models have also been developed and studied to understand a financial system’s contagion risk or the “domino effect” of banks taking down other banks. Although these analyses are valuable, they are not exhaustive and present researchers with two limitations. First, the target and explanatory variables or financial signals must be selected a priori from a large set of economic variables. Second, these methods have difficulty identifying and representing the data’s nonlinear, time-varying, and multidimensional relationships [2,21]. Our study introduces a novel methodology that addresses these limitations and enhances model explainability without sacrificing predictive power.

Financial stability, entropy, and information theory are interconnected concepts that provide a unique lens through which to analyze the resilience of financial systems. Financial stability refers to the robustness of financial institutions and markets, ensuring that they can withstand economic shocks without significant disruption [8]. Entropy, a measure of disorder or uncertainty, can be applied to financial markets to quantify the unpredictability of asset prices or economic indicators [6]. Information theory, particularly the concept of entropy, helps assess the efficiency of information dissemination in financial markets. During a financial crisis, high entropy in market signals may indicate a loss of information efficiency, where price movements become less predictable and more chaotic, leading to increased risk and instability. The application of these concepts to our analysis provides the framework for identifying the most influential subset of financial signals or variables that can predict these crises. Selecting the most influential variables that precede financial instability enables researchers to better understand how information asymmetry and market inefficiencies contribute to systemic risk, providing valuable insights into maintaining financial stability in volatile conditions.

Research focused on identifying the influential features that precede a period of increases financial risk or instability has taken many paths. Secrist [22] first analyzed national banks’ balance sheets to identify the metrics that define whether an institution would prosper or fail. He recognized the importance of interbank relationships in financial performance by incorporating ratios that compared the balance sheet totals of non-failing and failing banks to understand the characteristics that contributed to market volatility. Martin [23] introduced probit and logit multivariate regression models to identify banks at risk of losses that would result in a negative net worth. Frankel and Rose [19] applied regression methods to macroeconomic data to examine currency crashes in developing economies and characterized the contributions of foreign direct investments and debt to these failures. Bussiere and Fratzscher [24] proposed using a multinomial logit model as part of an EWS that recognizes the distinction between stable and precarious economic periods to predict financial crises in emerging markets. Their logit model addressed Martin’s [23] findings that identified a difference in the relationships between economic indicators before and after a financial crisis, where these independent variables required a stabilization period before the pre-crisis statistical relationships between dependent and independent variables resumed. Following the 2007–2009 financial crisis, Brownlees and Engle [25] introduced the SRISK measure to define the contribution of capital shortfalls to systemic risk as a function of a firm’s size, leverage, and potential loss of equity value in a market decline. While incorporating information on capital shortfalls, SRISK also represents the significance of individual institutions in the stability of financial markets, as the authors contend that aggregating SRISK provides a leading indicator of financial stress. While significant, all the above methods only focused on representing linear relationships between institutions and financial stability.

Network-based financial early warning systems are designed to predict financial crises by analyzing various indicators and data points. These predictions and the variables that inform their outcomes can then be used to inform policy interventions to mitigate or prevent the crisis. The addition of network models to EWS builds upon the importance of interbank relationships identified by Secrist [22] and relates to the study of the spread of contagion in epidemiology or cascading component failures in systems engineering. Current financial network models are based on Eisenberg and Noe [8], in which debt obligations define the strength of relationships (edges) between different banks (nodes). An economic shock proportional to the strength of the relationships between nodes is applied to the network, propagating along the edges, thereby decreasing the value of the affected bank’s debt obligations [26]. The propagation of the economic shock may be intensified when it results in a fire-sale activity in which a bank sells the shock-affected debt it lent on the market to access cash and reinforce its position [27]. While this action is locally stabilizing for the seller, it further destabilizes the network for other banks holding the same debt obligation by increasing the available supply of debt or shares for sale on the market. Debt obligation relationships between banks in a financial network define the strength of the propagated economic shock. If a severe financial shock initiates a fire-sale event, it could cause the failure of multiple banks owing to insolvency, where the banks cannot fulfill their debt obligations to each other. Owing to the network’s interconnectedness, the insolvency of one bank and the declining value of debt obligations can result in the failure of other banks in the network with related financial liabilities [11], resulting in financial contagion.

Advancements upon Eisenberg and Noe [8] have worked to integrate various computational techniques such as neural networks, genetic algorithms, Shapley and Aumman–Shapley values, and ML to improve the capabilities of financial early warning systems. Hsieh [28] applied genetic algorithms and neural networks to develop a financial early warning system for the Taiwanese banking industry. While their results demonstrated an improvement in predictive accuracy of 13% over other methods, their model was not interpretable, which limited its policy applications. Staum et al. [29] applied Shapley and Aumann–Shapley to financial network models to demonstrate how to perform systemic risk attribution. Their work provided valuable contributions to the basic theory of systemic risk attribution but required a priori knowledge of the feature’s importance, which may not effectively identify novel threats to financial stability. Samitas et al. [30] applied machine learning methods to significantly improve the predictive accuracy of network-based financial early warning systems for policymakers and investors with an effectiveness of 98.8%. The authors’ work demonstrated the utility of using machine learning algorithms in conjunction with network analysis to predict contagion risks. While impressive, their model may have been prone to overfitting owing to its high predictive accuracy and model training on the entire dataset.

Understanding financial contagion and, by extension, financial stability is complicated by conflicting messaging regarding how financial stability should be measured. The study of financial stability is broad, and researchers must consider everything from the impact of relationships between financial institutions to the variability and timing of financial markets when selecting explanatory variables for their models [31]. Financial contagion can propagate via multiple channels because of institutions’ dependence on the same funding sources [12]. However, selecting the most influential variables contributing to the spread of contagion during a specific period is challenging because of the curse of dimensionality [32]. Feature selection aims to counter this challenge by selecting the optimal subset of variables related to the phenomena of interest by removing irrelevant and redundant variables to improve model performance [33,34]. The feature selection problem is not limited to financial stability but also occurs in other subject areas. Examples include biostatistics [35], automotive engineering [36], healthcare [37], and computer vision [38].

Researchers tend to employ one of three traditional feature selection methods—filters, wrappers, or embedded methods [39]—to identify the most influential variables of a model and address the curse of dimensionality. When working with high-dimensional feature spaces such as financial markets, the multitude of choices makes selecting the correct subset of variables for a model a daunting challenge. Filter methods use specific criteria to select a subset of variables. Wrapper methods employ an ML model to select variables that improve the model’s measure of fit. Embedded methods use algorithms for feature selection and incorporate penalization to limit overfitting [34]. Each feature selection method has advantages and disadvantages. Although effective on smaller datasets, these traditional feature selection methods become challenging to implement computationally as the number of variables and observations increases [40].

Filter methods are employed as a data preprocessing step, where a relevant subset of variables is identified based on a priori statistical criteria. Common filtering methods employed for machine learning applications include correlation-based filters (univariate and multivariate) [41], consistency-based filters [42], information gain [43], relief-based algorithms [44], Fisher score [45], minimum redundancy [46], and maximum relevance [47]. Although computationally easier to employ, filter methods tend to yield less accurate predictive results [46].

Alternatively, wrapper methods utilizing machine learning models, such as support vector machines [48], K-nearest neighbors [49], and linear discriminant analysis [50], can also be an effective means of feature selection for high-dimensional data. The downside of wrapper methods is that they are computationally expensive owing to the recursive nature of the model training required to identify the optimal subset of variables [51].

Embedded methods incorporate the characteristics of filters and wrappers by embedding the feature selection process into the machine learning algorithm during training. This embedded characteristic allows this selection method to be more computationally efficient at the expense of predictive accuracy [52]. Common embedded method approaches include regularization (lasso, ridge, elastic nets) [53] and tree-based methods, such as random forest [54].

ML methods provide an opportunity to better define and understand the relationships that contribute to contagion risks and financial stability, particularly when dealing with multidimensional data and capturing nonlinear relationships [55]. Liu et al. [56] proposed using machine learning models, specifically random forest, gradient-boosted trees, and ensemble methods, to capture these nonlinear relationships and improve the performance of popular probit and logit regression methods. Ensemble methods enable researchers to combine multiple ML models, referred to as base estimators, to leverage their unique advantages and improve overall performance and robustness. Random forests are a type of ML ensemble that aggregates the output of multiple decision trees to improve the prediction accuracy, handle large datasets, and strengthen the model against outliers and noisy data [54]. Gradient-boosted trees are another type of ML ensemble decision tree method that incorporates the practice of boosting, where each sequential decision tree in the ensemble corrects upon the mistakes made by previous iterations and is highly effective at capturing complex patterns [56]. The effective and efficient use of these methods can empower researchers and policymakers to better understand how these relationships may change, and even cease to exist before and after financial crises.

Appreciating the hesitation of some decision-makers to utilize machine learning models owing to their lack of explainability, Liu et al. [56] also incorporated the use of Shapley-based methods to explore the causal relationships between macroeconomic variables, such as gross domestic product, and financial crises. This research builds upon their contributions by using SHAP and ensemble methods to create an explainable linear model that examines an individual firm’s contributions to the network’s stability. Improvements in SHAP over other feature selection methods enable our proposed framework to be implemented whenever significant shocks occur in the market [16]. As Martin [23] noted, there can be a significant departure in the relationships between economic variables before and after a shock, with some relationships no longer existing. Where Liu et al. [56] assume that the identified causal relationships remain, our framework accepts that these relationships may change and aims to identify the most influential variables over a specific period.

Compared with traditional feature selection methods, SHAP has proven to be more effective and efficient for identifying the most representative subset of variables while minimizing the use of computing and memory resources [16]. When used within the Shapley regression framework, Shapley values enable the discovery and expression of previously unknown relationships and functional forms similar to linear models [3]. Previous Shapley early warning system applications have been limited to providing insights into the black-box nature of the ML model’s output by demonstrating the impact of independent variables on the predicted response [57]. The ability to communicate these complex relationships in an explainable manner is essential for communicating technical concepts to audiences who may not be familiar with the research area. Integrating ensemble methods, SHAP, and modified Borda counts [15] enables the ranking of the feature importance vectors of the different ensemble base estimators along a standard criterion for comparison. A subset of the ensemble’s Borda-ranked variables can then be used to train a linear regression model representing the diversity of network structures between firms to obtain an explainable algorithm and prediction.

The proposed method also serves to limit model overfitting. An overfitted model refers to a high-variance model that has learned both the necessary signal and irrelevant noise of the training data, resulting in poor model generalization or performance when applied to new data [53]. Overfitting can occur when the model is excessively trained on the same training data or when it is overly complex and incorporates too many predictive variables. Working with highly dimensional datasets, such as that utilized in this study, can increase the chance that the researcher will select variables that perform well on the training data but generalize poorly when applied to a new dataset. Implementing ensemble methods for feature selection can prevent overfitting by reducing the complexity of the model by prescriptively limiting the number of predictive input variables [58].

This study contributes to the EWS literature by proposing a method to identify novel threats to financial networks that incorporates historical information from past financial crises and represents nonlinear, time-varying, and multidimensional relationships in capital markets [2]. Coulombe et al. [57] demonstrated that the ability of machine learning to capture nonlinearities is a critical advantage that improves macroeconomic predictions, especially when macroeconomic uncertainty is high and financial conditions are poor. Additionally, Alessi and Detken [1] demonstrated the ability of machine learning methods to identify influential variables as they relate to risks to financial stability, particularly during periods where “asymmetric information spurs excessive risk-taking and moral hazard.” We provide insight into the black-box nature of ML methods by using the selected variables to train a more parsimonious and explainable linear model [59]. These characteristics enable the proposed method to address the stated EWS limitations by enabling a priori feature selection and representing complex relationships in an explainable manner.

## 3. Material and Methods

### 3.1. Data

Our research uses supervisory financial data describing the Global Systemically Important Banks (G-SIBs) collected from the Federal Reserve (FR) Y-9C Consolidated Financial Statement for Holding Companies, FR Y-15 Banking Organization Systemic Risk Report, and correlated quarterly stock market share prices between the G-SIBs from 2017 to 2022. Evidence shows that aggregating financial data over multiple periods reveals the underlying structure of the financial network [60]. The data consist of approximately 3160 variables and 1800 observations. The Federal Reserve’s financial reporting quarters, represented as Q1, Q2, Q3, and Q4, were added to the data to represent the time dimension. Each quarterly correlated stock price corresponds to a pair of G-SIB variables annotated by including a “one” or “two” to identify whether the data correspond to the first or second bank in the pair. To limit overfitting of the ML models [53], the data were partitioned into training, validation, and test sets comprising 70%, 15%, and 15% of the observations, respectively. The training data were used to train the model, the validation data were used to tune the feature transformations and model hyperparameters, and the test data were used to test the model on out-of-sample data. Subsequently, a natural log transformation was applied to the final subset of the selected variables used to train the linear model to address positive skewness. The correlation of the stock price between the two banks over the Federal Reserve quarterly reporting period is calculated using Formula (1) [61]. Here, $r_{xy}$ represents the strength of the correlation between the daily stock prices of bank x and bank y over the reporting quarter.

(1) $$r_{xy}=\frac{n\sum xy-(\sum x)(\sum y)}{\sqrt{\left[n\sum x^{2}-{(\sum x)\;}^{2}\right]}\;\sqrt{\left[n\sum y^{2}-{(\sum y)\;}^{2}\right]}}$$

The correlated quarterly stock prices serve as proxies for bank exposure and financial market interdependence [62]. Higher values represent the likelihood that a shock to one bank in the network will affect other banks in that network. Evidence shows that asset return correlations in financial markets increase during financial crises [63]. The target-correlated quarterly stock prices account for financial risk and market participants’ reactions to adverse scenarios. The use of correlated quarterly stock prices as a proxy is not inferior to interbank exposure data and accounts for the indirect relationships between market participants [26]. Additionally, linear correlation values are used in this study because of their importance in modern portfolio theory [64] and financial time series [65] when optimizing the portfolio balance of high-risk, high-return stocks and low-risk, low-return stocks to determine the most efficient frontier. Other measures of market interdependence could be incorporated into the proposed method to account for the aforementioned disagreement on how financial contagion can and should be measured.

Following training, the performance of the sequential neural network, support vector regression, extreme gradient-boosted tree, and random forest models was assessed using mean absolute error (MAE), mean squared error (MSE), and root mean squared error (RMSE) on the unseen test dataset. Similar FR Y-9C, FR Y-15, and correlated quarterly stock price data from 2021 to 2023 were used for the March 2023 Silicon Valley Bank, out-of-sample, example. The example data consisted of 640 observations representing the variables selected by the proposed method.

### 3.2. Method Overview

In order to take advantage of advances in machine learning to provide early warning of instability, we developed a novel data-driven method to enable the construction of a network model of the financial system that captures the strength of relationships between financial entities. Estimating the strength of the relationships between these entities requires the use of a linear regression model generated through a novel feature selection method that leverages Shapley values and modified Borda counts combined with statistical and machine learning methods. The result is a low-dimensional, explainable statistical model capable of generating credible estimates of the strength of these relationships.

**Figure 1:**
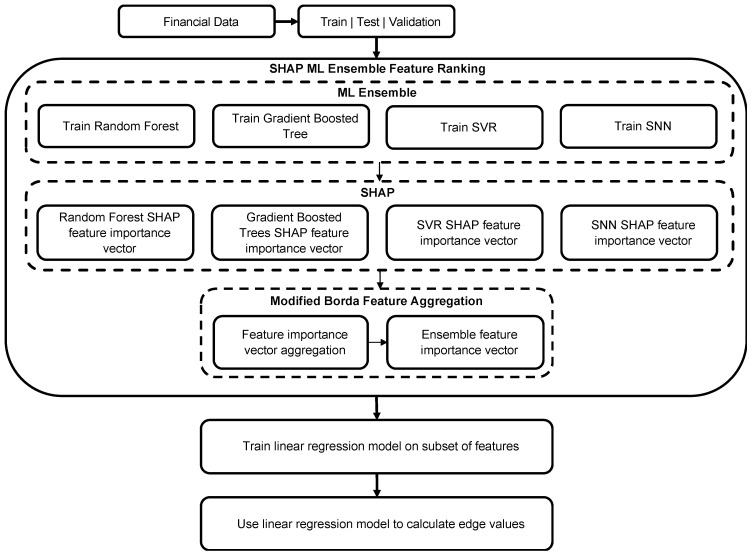
Proposed method to identify the subset of influential variables using ML ensemble methods, SHAP, and the modified Borda count method [66].

The proposed methodology is shown in Figure 1. The financial data were first partitioned into separate training, testing, and validation subsets to prevent overfitting of the ML models. The training subset is used to separately train the RF, extreme gradient-boosted trees, SNN, and SVR models, which have been incorporated together as an ensemble. The grouping of models as an ensemble enables us to take advantage of each method’s distinct abilities to identify unique patterns in the data. The validation and test subsets were used to tune the model parameters and test the model on out-of-sample data.

### 3.3. Model Training

RF, gradient-boosted trees, SVR, and SNNs were employed in this study owing to their documented efficacy in the financial network analysis literature [66,67]. Decision tree-based machine learning methods are applicable across a wide range of disciplines and applications, as they can address complex nonlinear and non-additive relationships without requiring specific prior knowledge about the functional form of the relationship under investigation [67]. SVR methods are particularly advantageous when working with nonlinear data, and the objective is to predict continuous outcomes with high accuracy [4]. SVR is an extension of support vector machines and applies principles of margin maximization to regression outcomes to develop models that are robust and capable of managing nonlinear relationships. SNNs are proficient in tasks that involve time series or sequential data. SNN architectures have been successful in predicting continuous outcomes where temporal sequences are significant because they can maintain information on time and capture dynamic temporal behaviors [28,67,68]. Ensemble methods have demonstrated superior accuracy in predicting financial distress. Hallajian et al. [33] proposed an ensemble feature selection method for binary classification. Niu et al. [68] utilized an ensemble of deep learning models, such as SNNs, to capture and represent the nonlinearities inherent in financial time series. Krauss et al. [67] employed an ensemble of deep learning networks, gradient-boosted trees, and RF to identify statistical arbitrage opportunities in the S&P 500.

There are two main types of RF models: random forest regressors and random forest classifiers. The random forest regressor is used in this study to predict continuous numerical values, whereas the random forest classifier is used to predict categorical or discrete classes. Due to our desire to predict the quarterly stock correlation coefficient between two G-SIBs, a continuous value, we used the random forest regressor.

The random forest regressor is used to identify patterns in the training subset of the data during a process called training. To train the model, we first define the number of estimators that we intend for our defined forest and initially set other tunable parameters, referred to as hyperparameters, that optimize the performance of the algorithm (maximum tree depth, minimum samples to split a node, etc.). Following training, we evaluated the model’s performance on the validation subset of data using MAE, MSE, and RMSE. Based on our initial results, we can fine-tune the hyperparameters using common techniques, such as cross-validation and grid search, on the validation data subset. Finally, we verify the ability of our random forest regressor to predict on unseen data by applying the trained model to our test data set and assessing MAE, MSE, and RMSE.

The development of the gradient-boosted tree model follows a similar training process, where the model’s hyperparameters are first initialized prior to applying the algorithm to the training subset of data. As the model iteratively assesses the data to identify underlying patterns, it calculates the negative gradient of the loss function to fit a decision tree and adds that weighted decision tree to the overall model to improve predictive accuracy. This process is repeated for a user-specified number of iterations before tuning the hyperparameters on the validation data subset to optimize the model performance. The trained model was then applied to the test data subset to verify its ability to perform on unseen data using the aforementioned metrics of MAE, MSE, and RMSE.

Training the SVR model starts by choosing the kernel type; common kernels include linear and polynomial kernels, and the radial basis function (RBF), which was used for this study. After kernel selection, the hyperparameters that define regularization, the margin of tolerance, and the kernel coefficient for RBF are established. We then train the model on the training subset of data, tune the hyperparameters to optimize performance on the validation subset, and then verify the ability of the model to perform on unseen data using the test data set.

The SNN model was prepared by first defining the neural network architecture by specifying the layer types, number of neurons per layer, and activation functions. The hyperparameters of the model were initialized to set the loss function, optimizer, and learning rate. The SNN model was then iteratively trained on the training subset of data using forward pass and back propagation to compute the loss and gradients to update the model weights. We then tuned the model’s hyperparameters to optimize performance on the validation data prior to confirming the model’s ability to perform on new data using our test data subset.

### 3.4. Ensemble Feature Ranking Aggregation

**Figure 2:**
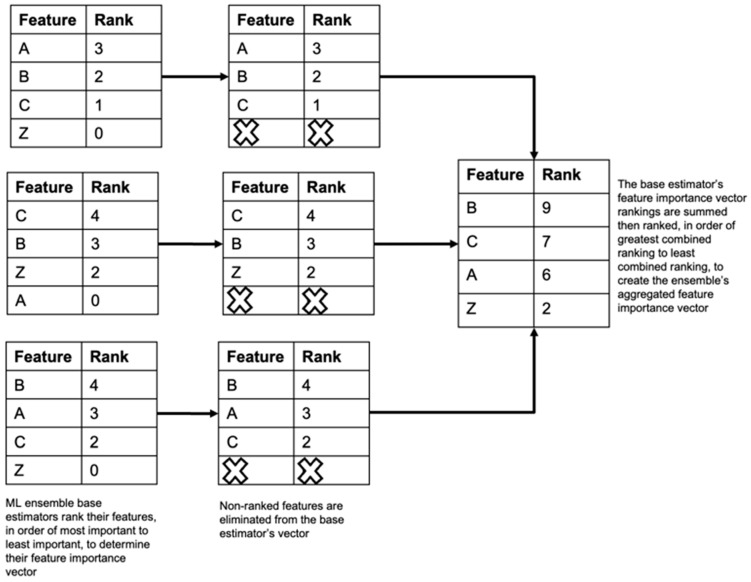
Modified Borda count feature aggregation. This figure illustrates the steps taken to implement the modified Borda count method to create the ML ensemble feature importance vector from the feature importance vectors of the individual base estimators.

Each model’s feature importance vector or feature ranking was then obtained and visualized using the Python SHAP package. SHAP provides an interpretation of the model’s feature rankings based on Shapley values, which we then use to rank features based on their impact on model predictions. Shapley values originate from cooperative game theory and have been adapted for use in machine learning to explain the outputs of complex models. They provide a method to fairly distribute the contribution of each feature to a model’s prediction [14]. The SHAP package was implemented by first training the desired models and applying the SHAP explainer function to the test data to calculate the Shapley values. The feature importance ranking was then calculated using the SHAP feature importance function. The model’s feature ranking, or feature importance, vector can also be visualized using the SHAP summary plot function. The feature importance vectors are aggregated using the modified Borda feature aggregation method shown in Figure 2 to create a combined feature importance vector representing the aggregated feature ranking of the ML ensemble’s base estimators.

Aggregating the feature importance vectors of the ensemble’s models, or base estimators, via the modified Borda count method shown in Figure 2 allows us to combine the strengths of each base estimator for feature selection and develop a linear regression model capable of generalizing well on unseen data. The modified Borda count method is a voting system used for ranking multiple options, which we have adopted to rank our ensemble’s models’ feature importance rankings along a common measure. After obtaining each model’s feature importance vector, we then assign points to each feature based on the feature’s ranked position. The top-ranked feature receives the maximum number of points based on the total number of ranked features, with each subsequently ranked feature receiving one less point. Unranked features receive zero points in the modified Borda count method. The feature’s points are summed across the ensemble’s contributing models, to determine the aggregated or global feature importance vector for the ensemble. The feature with the most points is the most important feature across the ensemble. The modified Borda count method provides a more flexible and robust approach to preferential voting, particularly in scenarios with varying numbers of candidate features or where the ensemble’s model may not have ranked all features [15].

The aggregated ML ensemble feature importance vector was then used to enable an a priori or informed development of an explainable linear model for economists and government policymakers. The high-dimensional feature space of the problem, exceeding 3000 individual variables representing each G-SIB’s combined quarterly FR Y-9C and FR Y-15 reports, presents challenges for building explainable models that our method addresses. Each model’s feature importance vector, or feature ranking, was obtained via SHAP to understand the impact of the independent variables on the response feature. The computed additive SHAP value represents the mean marginal contribution of a feature across all possible combinations [14].

We refrained from prematurely removing highly collinear predictors to avoid inadvertent removal of the nonlinear relationships we wished to represent. Multicollinearity occurs when linear dependencies exist between explanatory variables [69]. Removing the linear relationships between the explanatory variables at the beginning of our process can result in the removal of influential nonlinear relationships. After identifying possible nonlinear relationships with our ensemble’s base estimators, we evaluated multicollinearity using variance inflation factors (VIFs) before fitting our final linear regression model [70].

### 3.5. Model Fitting

A separate linear model is fitted for each Shapley ranked subset of variables using forward feature selection, where an additional feature is added in the order of importance to create each additional subset [34]. The forward feature selection process was stopped once the MAE, MSE, and RMSE failed to improve with the addition of each new feature to the model. The stopping criterion can also be represented visually as a reduction in slope, or an inflection point in error vs. the number of feature plots, where the evaluated error per feature decreases with the addition of each new feature to the subset. This process was repeated for each of the ensemble’s base estimators to develop the final linear regression model, as depicted in Table 1.

Evidence of multicollinearity among the Borda selected variables should be evaluated to help the linear regression model generalize on unseen data. Not reducing multicollinearity among independent variables can lead to inconsistent predictions owing to inaccurate model coefficient estimates [69]. Among the selected variables of the linear regression model, FR Y-15 RISIM334: SECURITIES FINANCING TRANSACTION (SFT) EXPOSURES: GROSS SFT ASSETS, and RISIM388: PAYMENTS MADE IN THE LAST FOUR QUARTERS: UNITED STATES DOLLARS (USD) have a high level of multicollinearity, with a Pearson correlation coefficient (R-value) of 0.88. To understand the impact of these variables, we computed the VIF and evaluated the performance of the model with and without each feature. As a standard practice, a feature with a VIF > 5 should be considered a candidate for removal [69]. RISIM334 and RISIM388 have VIFs of 6.34 and 6.73, respectively.

An evaluation of the linear model’s MSE using K-fold cross-validation, with and without RISIM334 and RISIM388, confirmed that removing the variables had a negligible impact on model performance. Cross-validation is a resampling method that draws repeated subsets from the training data to aid in model selection. The method is particularly useful when attempting to train a model on data with limited observations, but can be computationally expensive owing to repeatedly training multiple versions of a model to determine the best version [69]. In this instance, we utilized K-fold cross-validation (k = 10) to limit the computational impact of repeated training when selecting between the different models, with and without the collinear variables, by evaluating the MSE of each model across the 10 samples or folds. The average MSE across the 10 samples was 0.074, compared with the original model’s MSE of 0.092, confirming the removal of the two collinear variables. The removal of these two variables resulted in our final linear model, as shown in Table 1.

### 3.6. Network Construction

**Figure 3:**
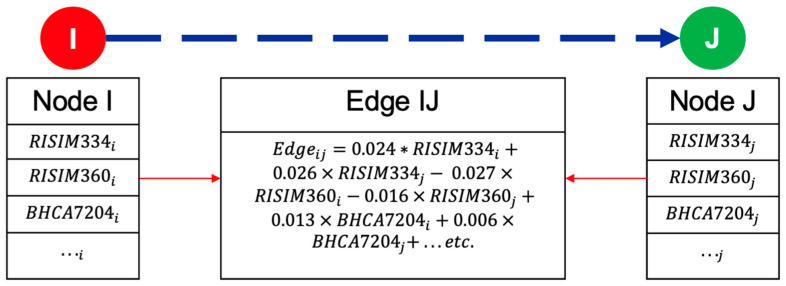
Construction of financial network EWS. The figure illustrates the construction of a financial network model, where the nodes represent financial institutions with their represented variables from the FRB Y-9C and Y-15 reports, and the linear regression model is used to calculate the edge values. RISIM334 is the Securities Financing Transaction (SFT) Exposure: Gross SFT Assets, RISIM360 is the Over the Counter (OTC) derivatives with unaffiliated financial institutions that have a net positive fair value: potential future exposure, and BHCA7204 is the Tier 1 leverage ratio. “…” represents the additional predictive variables from the financial institution pair, described in Table 2, needed to calculate the edge value.

The proposed financial network EWS was constructed by representing each financial institution and its associated selected input variables with a node, as shown in Figure 3. The edge values, which represent the quarterly correlated stock values between the two nodes, were calculated using the trained linear mode (Table 1). Financial system shocks were modeled by altering one of the node’s variables and recalculating the weights of the edges using a linear regression model. After updating the weights, a shock can be propagated by changing the associated stock price of the G-SIB node and observing the propagation of the change throughout the network. Following the standard convention of market correction, a significant change was identified as a greater than 10% decline in the G-SIB’s stock price [71]. Additional contagion insights were obtained by reviewing quarterly correlated stock prices or edge weights between nodes. In particular, an increase in correlation values among the nodes indicates contagion conditions and a financial network that is affected more by the actions or events of one of the banks in the network [27].

## 4. Results

The comparative performance of the proposed model, using variables identified by the proposed methodology, compared to other filter, wrapper, and embedded feature selection methods, is detailed in Table 2. The comparative linear models were evaluated using out-of-sample test data, met the assumptions for linear regression [69], and were consistent across the performance metrics. Linear regression models that relied on variables selected using recursive feature elimination, consistency-based filtering, minimum redundancy, maximum relevance, and support vector regression feature selection outperformed the proposed method at the computational expense of including 70 or more variables. Additionally, models that use an excessive number of variables are prone to overfitting [69].

The proposed model, utilizing variables selected by the ML ensemble, outperformed linear regression models informed by forward, backward, univariate filter correlation, information gain, Fisher score, random forest, extreme gradient-boosted trees, and sequential neural network feature selection methods. When employing univariate filter correlation, information gain, Fisher score, and forward and backward feature selection, the proposed number of variables (k_variables) must be defined a priori as an input hyperparameter. For comparative purposes, the “k_variables” input hyperparameter was set to the same number of variables identified by our proposed method. However, the “k_variables” hyperparameter can also be defined by implementing unsupervised learning techniques, such as K-means clustering, but this choice results in a similar limitation of having to define the number of clusters hyperparameter a priori [53].

## 5. Application: Silicon Valley Bank Failure

We applied our trained linear model to quarterly FR Y-15 and Y-9C data, which represent approximately one year before the failure of the Silicon Valley Bank in March 2023, to validate our proposed methodology. The data consisted of 640 observations from 2021 to 2023, representing the 14 variables identified using the proposed feature selection method. The data used in this example are separate from those used to train, validate, and test the initial linear regression model described in Table 1.

**Figure 4:**
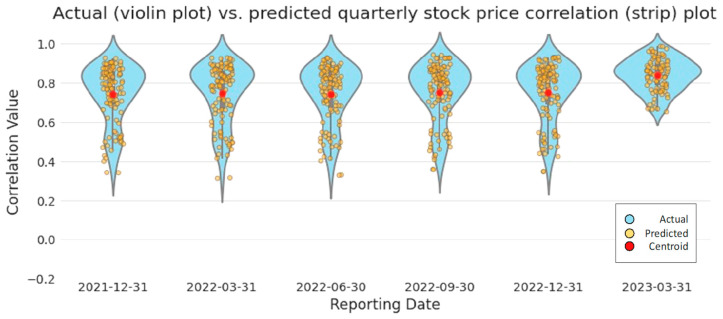
Actual versus predicted quarterly stock price correlation plot. The distribution of the actual quarterly stock price correlation values is displayed as a blue violin plot, with the predicted and centroid values overlaid in yellow and red, respectively. (Color).

A plot of the actual vs. predicted correlated quarterly stock Figure 4, as well as measures of fit (MAE, MSE, and RMSE; Table 3), demonstrate the ability of our trained model to generalize on the unseen data. The figure consists of a blue-shaded violin plot that shows the distribution of the actual quarterly correlated stock values, with wider portions representing a greater number of points with similar values. In contrast, the predicted quarterly correlated stock values are plotted as yellow strips.

**Figure 5:**
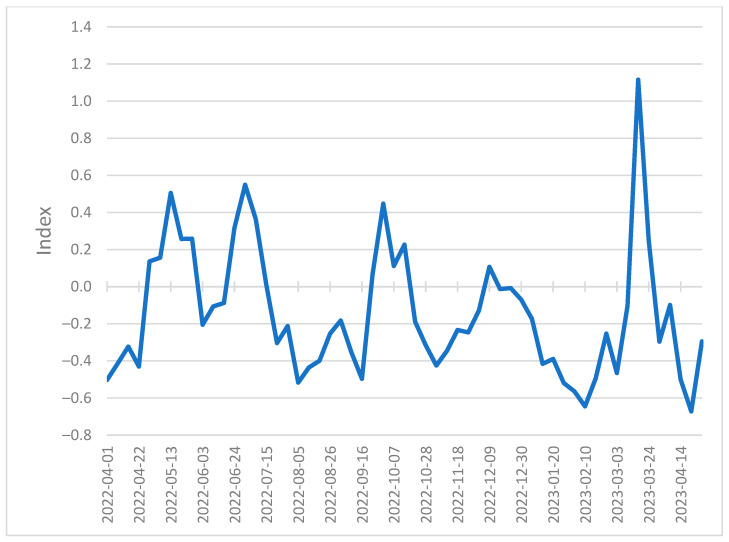
Federal Reserve Bank of St. Louis Financial Stress Index (Weekly, Not Seasonally Adjusted) identifies periods of high stress in financial markets over time.

We observe that the predicted quarterly stock price correlations appear consistent with the actual values, increasing in March 2023 during the Silicon Valley Bank failure, representing the increased risk to financial markets. Our model results in Figure 4 correspond to the St. Louis Federal Reserve Bank Financial Stress Index (FSI) Figure 5, which is consistent with documented observations of stock price correlations increasing during periods of high financial stress [63]. During a period of increased stock price correlations, a change in the stock price of one financial institution increasingly affects the stock prices of other financial institutions in the network, representing an increased likelihood of contagion [27].

**Figure 6:**
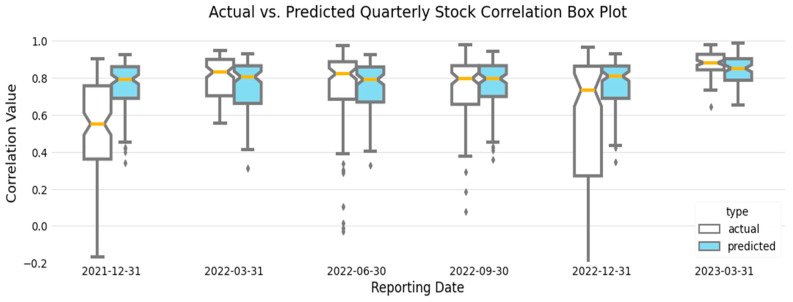
Actual vs. predicted quarterly stock correlation boxplot depicts the distribution of actual vs. predicted values over quarterly reporting periods. (Color).

Box plots of our actual quarterly stock correlation values and predicted quarterly stock values Figure 6 provide a more detailed view of the actual vs. predicted value distribution. One noticeable difference is the lower-than-expected actual quarterly stock correlation values in December 2021. These lower values correspond to a period of lower financial stress in the FSI at the beginning of January 2022. However, this period of lower actual quarterly stock correlation values is not reflected in our predicted results, most likely because our linear model generalizes across the associated training data. The FSI and our resulting plots Figure 4, Figure 5 and Figure 6 show a period of relatively low financial stress prior to peaking during the collapse of several financial institutions at the end of March 2023. This pattern of relative calm before the storm is also demonstrated in the centroid values plotted in red in Figure 4. We observe quarterly correlated stock values that coalesce at approximately 0.750 before peaking at 0.841 at the end of March 2023.

**Figure 7:**
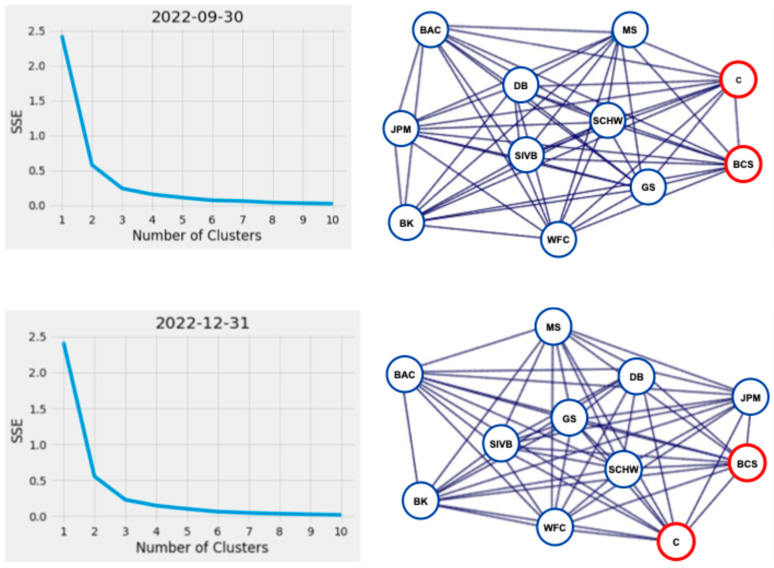

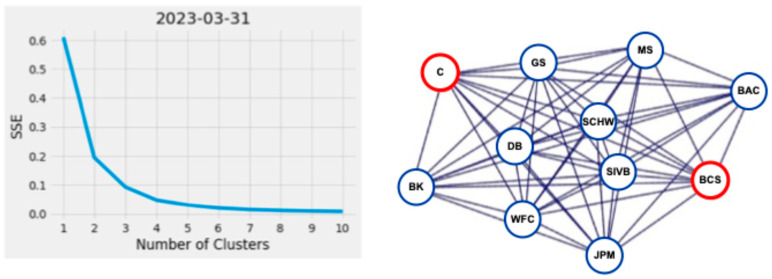
Knee and graph plots showing the reduction in SSE as the number of clusters increases with the corresponding network plots from the same quarterly reporting period, showing the financial institutions (nodes) driving the creation of the three clusters from September 2022 to March 2023. Key: Bank of America (BAC), Barclays (BCS), Bank of New York Mellon (BK), Citigroup (C), Deutsche Bank (DB), Goldman Sachs (GS), Morgan Stanley (MS), JP Morgan (JPM), Silicon Valley Financial Group (SIVB), Charles Schwab (SCHW), Wells Fargo (WFC). (Color).

Further inspection of actual versus predicted quarterly stock price correlation values (Figure 4) shows three clusters of values representing the corresponding correlated stock values between financial institutions per reporting period, except at the end of March 2023, when the clusters visually appear to combine into one large group. The contraction of these values is also shown in Figure 6 by the reduction in the interquartile range in March 2023. Cluster analysis of the financial network represented in our sample data using K-means standard square error vs. number of clusters plot, programmatically using Python’s KneeLocator() function, and utilizing the network analysis (NetworkX) community methods, confirmed three consistent groupings of financial institutions across all reported periods, including March 2023, identified as BCS, C, and other institutions in Figure 7. Despite visually appearing to be combined into one group at the end of March 2023, the three identified clusters remained programmatically. In the event of a stock price change initiated by a G-SIB in the financial institution cluster defined as [BAC, BK, DB, GS, JPM, MS, SCHW, WFC], the stock prices of clusters [BCS] and [C] would be affected the least.

## 6. Discussion

Applying the linear model to the out-of-sample Silicon Valley Bank, U.S. March 2023, financial reporting data demonstrate the utility of the proposed method in post mortem financial analysis. While the feature coefficients of a linear model can tell us how much the output of a model changes when the associated feature changes, they do not do a great job of conveying the overall importance of the variables when determining the predicted response. Utilizing Shapely values allows us to better understand the impact of changing the selected variables in the context of the output of the linear model and the distribution of the values of the selected variables [14].

**Figure 8:**
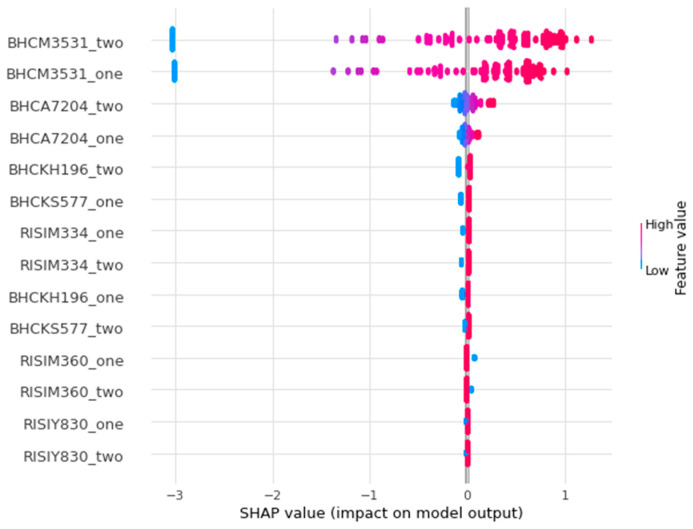
SHAP feature importance plot showing the relative importance of BHCM3531: U.S. Treasury Securities and BHCA7204: Tier 1 Leverage Ratio between GSIB pairs, identified as “_one” for GSIB one and “_two” for GSIB two. BHCA7204 is Tier 1 Leverage Ratio, BHCKH196 is Unsettled Transactions (Failed Trades)(Allocation by Risk Weight Category 100%)(Bank Holding Company Consolidated), BHCKS577 is Risk-Weighted Assets by Risk-Weight Category (Allocation by Risk-Weight Category 625%), RISIM334 is Securities Financing Transaction (SFT) Exposures: Gross SFT Assets, RISIM360 is Over the Counter (OTC) Derivatives with Unaffiliated Financial Institutions that have a Net Positive Fair Value: Potential Future Exposure, and RISIY830 is Other On-Balance Sheet Exposures: Other On-Balance Sheet Assets. (Color).

When this concept is applied to the predicted outcomes of the SVB financial data, we can observe the impact of U.S. Treasury Securities, identified as BHCM3531_one and BHCM3531_two, during the March 2023 financial crisis. The role of U.S. Treasury Securities on the Shapley feature importance plot Figure 8 is consistent with that reported in financial media outlets [72]. The Shapley plot shows the relative importance of the feature upon the linear model’s predicted output, with features higher on the *y*-axis being more impactful and those lower on the *y*-axis being less impactful. The feature’s values are color-coded in gradients from red to blue, with reddish colors indicating higher feature values and bluish colors representing lower values. When SVB depositors began withdrawing their deposits, they had to heavily discount their longer-term held bonds to cover the withdrawals and compete with shorter-term securities. SVB’s collapse demonstrated that the traditionally risk-free asset of U.S. Treasury Bonds is only risk-free if held to maturity, exposing SVB to interest-rate risk that resulted in a fire sale [72].

The proposed method facilitates the development of an EWS by providing a parsimonious model that represents the complexities of relationships in financial networks. Our results show that ML methods can accurately estimate the “complex, nonlinear, time-varying, and multidimensional” [2] relationships between independent input and response variables. Applying our methodology to the U.S. March 2023 financial reporting data demonstrates that our method can identify novel market threats while incorporating historical information from past financial crises.

The integrated use of Shapley values and modified Borda counts, in combination with methods from statistical and machine learning, provides economists, policymakers, and financial analysts with a method to identify evolving threats to complex financial networks that may not conform to existing frameworks, while incorporating the lessons learned from past financial crises. The novel feature selection method identified several influential variables, such as the leverage ratio [73], which are standard indicators of systemic financial risk. When applied to financial regulatory reporting data, the model also demonstrated the impact of U.S. treasuries, typically considered safe assets, during the March 2023 Silicon Valley Bank financial crisis.

There are limitations that one should be aware of before implementing the proposed method. This process is not suitable for all types of data, such as unstructured or non-labeled datasets, and may require additional preprocessing and transformations to achieve optimal performance. In our study, a significant level of effort was required to preprocess the data prior to training the ensemble’s models and obtaining their feature rankings. Additionally, users should be wary of prematurely declaring causal relationships without supplementary evidence. Therefore, subject matter expertise is essential because the proposed method only reveals the existence of a relationship. Therefore, context and in-depth knowledge should be considered when assessing whether a relationship is causal. Finally, our study was only applied to U.S. markets and may not be as effective in different financial systems. While it is probable that the proposed methodology and the resulting financial EWS can be modified to represent other markets, such as the London Stock Exchange or the Tokyo Stock Exchange, this should not be assumed without further analysis.

We demonstrate the representation of complex financial relationships in relatively simple and explainable linear models with only a modest increase in error. Our linear model is then used to update the edge value weights in our financial stability EWS, where the nodes represent the G-SIBs and the edge values represent the correlated quarterly stock prices. This method and the resultant model may benefit economic, public policy, and financial regulatory researchers who aim to infer relationships from highly dimensional, infrequently reported financial data.

## 7. Conclusions

The proposed method addresses some of the challenges in developing a financial network EWS by capturing the time-varying, nonlinear relationships between G-SIBs. This task was accomplished using an ensemble of ML models, SHAP, and modified Borda count methods to identify the most influential subset of variables. This method efficiently reduced the feature space from over 3000 variables, representing the FR Y-15 and Y-9C reports of the G-SIB pair, to 14. The selected subset of variables was then used to train an explainable and parsimonious linear regression model that outperformed the ML ensemble’s base estimators and was used to calculate the edge weights, or quarterly correlated stock prices, of the financial EWS network model. We then demonstrated the utility of the proposed method by applying it to the March 2023 Silicon Valley Bank Crisis.

Possible areas of future exploration include an economic analysis of the SHAP feature importance vector results to identify causal relationships based on statistical significance, assessing the effectiveness of the proposed method in other financial networks, identifying the proper timeframes to implement the EWS framework before and after a crisis, expanding the training data to include non-G-SIBs and other macroeconomic data, incorporating the use of more robust correlation measures to represent market interdependence, and the inclusion of additional algorithms, such as long short-term memory models, into the ML ensemble.

## Figures and Tables

**Table 1:** Linear regression model coefficients.

Feature	Description	Coefficient
RISIM334_one	SECURITIES FINANCING TRANSACTION (SFT) EXPOSURES: GROSS SFT ASSETS (Bank 1)	0.024
RISIM334_two	SECURITIES FINANCING TRANSACTION (SFT) EXPOSURES: GROSS SFT ASSETS (Bank 2)	0.026
RISIM360_one	OVER THE COUNTER (OTC) DERIVATIVES WITH UNAFFILIATED FINANCIAL INSTITUTIONS THAT HAVE A NET POSITIVE FAIR VALUE: POTENTIAL FUTURE EXPOSURE (Bank 1)	−0.027
RISIM360_two	OVER THE COUNTER (OTC) DERIVATIVES WITH UNAFFILIATED FINANCIAL INSTITUTIONS THAT HAVE A NET POSITIVE FAIR VALUE: POTENTIAL FUTURE EXPOSURE (Bank 2)	−0.016
BHCA7204_two	TIER 1 LEVERAGE RATIO (Bank 2)	0.013
BHCA7204_one	TIER 1 LEVERAGE RATIO (Bank 1)	0.006
BHCKH196_one	UNSETTLED TRANSACTIONS (FAILED TRADES) (ALLOCATION BY RISK WEIGHT CATEGORY 100%) (BHC CONSOLIDATED) (Bank 1)	0.014
BHCKH196_two	UNSETTLED TRANSACTIONS (FAILED TRADES) (ALLOCATION BY RISK WEIGHT CATEGORY 100%) (BHC CONSOLIDATED) (Bank 2)	0.020
RISIY830_two	OTHER ON-BALANCE SHEET EXPOSURES: OTHER ON-BALANCE SHEET ASSETS (Bank 2)	0.031
RISIY830_one	OTHER ON-BALANCE SHEET EXPOSURES: OTHER ON-BALANCE SHEET ASSETS (Bank 1)	0.026
BHCM3531_two	U.S. TREASURY SECURITIES (CONSOLIDATED) (Bank 2)	0.030
BHCM3531_one	U.S. TREASURY SECURITIES (CONSOLIDATED) (Bank 1)	0.031
BHCKS577_one	RISK-WEIGHTED ASSETS BY RISK-WEIGHT CATEGORY (ALLOCATION BY RISK WEIGHT CATEGORY 625%) (BHC CONSOLIDATED) (Bank 1)	0.047
BHCKS577_two	RISK-WEIGHTED ASSETS BY RISK-WEIGHT CATEGORY (ALLOCATION BY RISK WEIGHT CATEGORY 625%) (BHC CONSOLIDATED) (Bank 2)	0.019

Note: A “_one” or “_two” identifies whether the feature corresponds to the first or second bank.

**Table 2:** Comparative performance of feature selection methods on unseen test data.

Feature Selection Method	Total Variables	MAE	MSE	RMSE
Random Forest Regression	15	0.247	0.105	0.324
**Proposed ML Ensemble Method**	**16**	**0.224**	**0.092**	**0.303**
Backward	16 *	0.23	0.091	0.302
Forward	16 *	0.233	0.093	0.305
Fisher Score	16 *	0.237	0.099	0.314
Information Gain	16 *	0.246	0.105	0.324
Univariate Filter Correlation	16 *	0.248	0.103	0.321
Extreme Gradient-Boosted Trees	18	0.239	0.099	0.315
Sequential Neural Network	40	0.225	0.095	0.308
Support Vector Regression	72	0.218	0.088	0.297
Recursive Feature Elimination	524	0.162	0.056	0.237
Maximum Relevance	887	0.147	0.068	0.261
Consistency-Based Filter	1744	0.148	0.068	0.261
Minimum Redundancy	1859	0.149	0.067	0.259

Note: The ensemble-selected variables in the linear regression model show the reported error of the proposed linear regression model compared to other linear regression models informed by alternative feature selection methods. The total number of variables (k_variables) for the annotated feature selection methods identified by (*) were defined a priori as a required hyperparameter. For comparison purposes, the k_variables hyperparameter was set to the same number of variables as utilized for the proposed method.

**Table 3:** Model performance on Silicon Valley Bank failure data.

MAE	MSE	RMSE
0.167	0.046	0.215

## Data Availability

Data supporting the findings of this study are available from the corresponding author upon reasonable request. The data were derived from the following resources available in the public domain: FR Y-9C and Y-15 data are available from the Federal Reserve Board of Governors, Federal Financial Examination Council, and National Information Centre at https://www.ffiec.gov/npw, accessed on 20 August 2022. G-SIB stock prices are available at Yahoo Finance at https://finance.yahoo.com/, accessed on 20 August 2022.
